# Association between acute kidney injury and serum procalcitonin levels and their diagnostic usefulness in critically ill patients

**DOI:** 10.1038/s41598-019-41291-1

**Published:** 2019-03-18

**Authors:** Kayeong Chun, Wookyung Chung, Ae Jin Kim, Hyunsook Kim, Han Ro, Jae Hyun Chang, Hyun Hee Lee, Ji Yong Jung

**Affiliations:** 1 Division of Nephrology, Department of Internal Medicine, Gachon University Gil Medical Center, Incheon, Korea; 2 Gachon University College of Medicine, Incheon, Korea; 3 Gachon Medical Research Institute, Incheon, Korea

## Abstract

Procalcitonin (PCT) is a useful marker for the diagnosis of systemic inflammatory response syndrome. In addition, PCT is affected by renal function. However, few studies have investigated the relationship between PCT and the development of acute kidney injury (AKI). Hence, we investigated whether serum PCT levels at the time of admission were associated with the development of AKI and clinical outcomes. A total of 790 patients in whom PCT was measured on admission to the intensive care unit (ICU) were analyzed retrospectively. We attempted to investigate whether serum PCT levels measured at the time of admission could be used as a risk factor for the development of AKI in septic and nonseptic patients or as a risk factor for all-cause mortality, and diagnostic usefulness of PCT was further assessed. Serum PCT levels were significantly higher in patients with AKI than in those without AKI (*P* < 0.001). After multivariable adjustment for clinical factors, laboratory findings, and comorbidities, PCT as a continuous variable showed a significant association with AKI (OR 1.006, 95% CI [1.000–1.011]; *P* = 0.035). However, PCT was not effective in predicting mortality. The cut-off value of PCT for the prediction of AKI incidence was calculated to be 0.315 ng/ml, with sensitivity and specificity of 60.9% and 56.9%, respectively. The odds ratios (ORs) from an equation adjusted for optimum thresholds of PCT levels for developing AKI with and without sepsis were 2.422 (1.222–4.802, *P* = 0.011) and 1.798 (1.101–2.937, *P* = 0.019), respectively. However, there were no absolute differences between the pre- and posttest probabilities after including the PCT value for AKI development. This study suggests that the PCT value was higher in AKI patients than in non-AKI patients, but PCT measurement at the time of admission did not improve the prediction model for AKI.

## Introduction

Acute kidney injury (AKI) occurs in 5 to 7% of all hospitalized patients^1^. With a frequency of 36 to 67%, however, AKI occurs more frequently in critically ill patients^2–4^. The development of AKI in patients in the intensive care unit (ICU) varies depending on their underlying etiology (e.g., sepsis, trauma, major surgery, or exposure to contrast medium), but once AKI occurs, the results are associated with significant morbidity and high mortality^4^.

To date, many studies have tried to prevent the occurrence of AKI and find biomarkers to predict AKI^3,5^. In recent years, various laboratory parameters, such as neutrophil gelatinase-associated lipocalin (NGAL), kidney injury molecule-1 (KIM-1), liver-type fatty acid binding protein (L-FABP), interleukin-18 (IL-18), and cystatin C, have been proposed as potential biomarkers of AKI^5–8^.

Procalcitonin (PCT) is a 116-amino-acid precursor of calcitonin with a molecular weight of 13,600 Da. PCT is undetectable, or only barely detectable, in healthy individuals. However, in cases of sepsis, PCT levels can fluctuate in the range of 10 to 100 ng/ml and are accompanied by elevations in cytokines such as tumor necrosis factor-α (TNF-α), interleukin-1 (IL-1), and interleukin-6 (IL-6)^9^. Recently, it has been reported that renal function is a major determinant of PCT levels, and thus, different thresholds should be applied according to impairments in renal function^10–13^. However, few studies have investigated the relationship between PCT levels and AKI.

Therefore, we investigated whether serum PCT levels at the time of ICU admission were associated with the development of AKI and clinical outcomes in critically ill patients.

## Results

### Baseline characteristics

**Figure 1:**
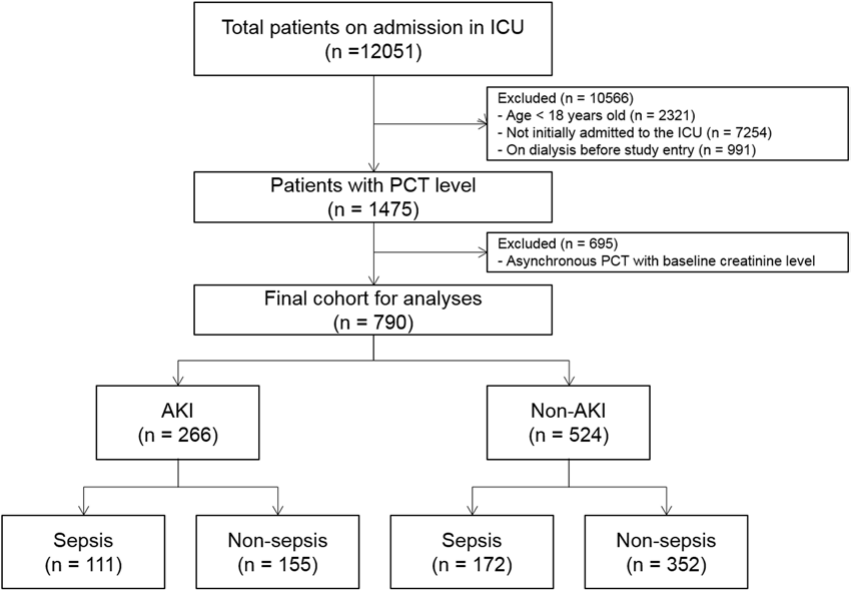
Cohort formation.

A total of 790 (478 males) of 12,051 screened subjects were enrolled as eligible study participants (Fig. 1). The mean age was 60.3 ± 17.1 years. Basal characteristics are described in Table 1. A total of 33.7% (266 of 790) of patients developed AKI. Furthermore, 41.7% (111 of 266) of patients with AKI and 32.8% (172 of 524) of patients without AKI had sepsis. AKI patients were more likely to have sepsis than those in the non-AKI group were (*P* = 0.014). These patients also commonly had chronic kidney disease (CKD), diabetes mellitus (DM), hypertension (HTN), chronic liver disease (CLD), and previous cardiovascular disease (CVD). Patients who developed AKI had significantly higher serum creatinine and hsCRP levels and lower blood pH than did patients who did not develop AKI. PCT levels in the AKI group were significantly higher than those in the non-AKI group (*P* < 0.001).

**Table 1:** Baseline characteristics.

	Total (N = 790)	AKI (N = 266)	No AKI (N = 524)	*P*
**Demographic**				
Age, year	60.3 ± 17.1	62.4 ± 16.1	59.3 ± 17.6	0.012
Male gender, n (%)	478 (60.5%)	159 (59.8%)	319 (60.9%)	0.764
**Medical history**				
CKD, n (%)	59 (7.5%)	39 (14.7%)	20 (3.8%)	<0.001
DM, n (%)	84 (10.6%)	59 (22.2%)	25 (4.8%)	<0.001
HTN, n (%)	133 (16.8%)	88 (33.1%)	45 (8.6%)	<0.001
CVD, n (%)	150 (19.0%)	86 (32.3%)	64 (12.2%)	<0.001
CLD, n (%)	27 (3.4%)	15 (5.6%)	12 (2.3%)	0.014
COPD, n (%)	33 (4.2%)	16 (6.0%)	17 (3.2%)	0.066
Asthma, n (%)	32 (4.1%)	18 (6.8%)	14 (2.7%)	0.006
**AKI risk factors**				
Sepsis, n (%)	283 (35.8%)	111 (41.7%)	172 (32.8%)	0.014
Transfusion, n (%)	360 (45.6%)	148 (55.6%)	212 (40.5%)	<0.001
Ventilator care, n (%)	520 (65.8%)	184 (69.2%)	336 (64.1%)	0.157
**Medications**				
RAS blockers, n (%)	76 (9.6%)	53 (19.9%)	23 (4.4%)	<0.001
CCB, n (%)	59 (7.5%)	38 (14.3%)	21 (4.0%)	<0.001
Beta-blocker, n (%)	64 (8.1%)	45 (16.9%)	19 (3.6%)	<0.001
Diuretics, n (%)	68 (8.6%)	45 (16.9%)	23 (4.4%)	<0.001
Inotropics, n (%)	543 (68.7%)	207 (77.8%)	336 (64.1%)	<0.001
Aminoglycoside, n (%)	40 (5.1%)	8 (3.0%)	32 (6.1%)	0.060
Vancomycin, n (%)	114 (14.4%)	41 (15.4%)	73 (13.9%)	0.575
Colistin, n (%)	22 (2.8%)	11 (4.1%)	11 (2.1%)	0.100
NSAID, n (%)	436 (55.2%)	134 (50.4%)	302 (57.6%)	0.053
Contrast, n (%)	63 (8.0%)	20 (7.5%)	43 (8.2%)	0.736
Chemotherapy, n (%)	6 (0.8%)	1 (0.4%)	5 (1.0%)	0.670
Laboratory findings*				
PCT (ng/ml)	0.3 (0.1, 3.6)	0.7 (0.1, 7.1)	0.2 (0.1, 2.7)	<0.001
Creatinine (mg/dl)	1.1 (0.8, 1.4)	1.3 (1.0, 2.1)	1.0 (0.7, 1.2)	<0.001
hsCRP (mg/dl)	1.6 (0.1, 11.1)	2.5 (0.2, 14.3)	1.3 (0.1, 9.5)	0.005
WBC (10^3^/mm^3^)	12.7 (9.1, 18.0)	13.1 (9.8, 18.5)	12.5 (8.9, 17.8)	0.116
pH	7.3 (7.2, 7.4)	7.3 (7.3, 7.4)	7.4 (7.2, 7.4)	<0.001
pCO2 (mmHg)	35 (28, 47)	35 (27, 52)	35 (29, 46)	0.208
SpO2 (mmHg)	70 (47, 101)	68 (43, 95)	72 (48, 103)	0.153
Severity of illness*				
SOFA score	6.0 (4.0, 8.0)	6.0 (4.0, 8.0)	6.0 (4.0, 8.0)	0.287

Abbreviations: AKI, acute kidney injury; CKD, chronic kidney disease; DM, diabetes mellitus; HTN, hypertension; CVD, cardiovascular disease; CLD, chronic liver disease; COPD, chronic obstructive pulmonary disease; RAS, renin-angiotensin-aldosterone system; CCB, calcium channel blocker; NSAID, nonsteroidal anti-inflammatory drug; PCT, procalcitonin; hsCRP, highly sensitive C-reactive protein; WBC, white blood cell; pCO2, partial pressure of carbon dioxide; SpO2, peripheral oxygen saturation; SOFA, Sequential Organ Failure Assessment. *Variables are expressed as the median and IQR.

### The development of AKI and serum PCT levels at the time of admission

Of the 790 patients, 266 developed AKI, with severity stages as follows: stage 1 (N = 172), stage 2 (N = 56), and stage 3 (N = 38). In the univariate analyses using PCT as a continuous variable, PCT at the time of admission was significantly associated with the development of AKI (odds ratio [OR; 1.007], 95% confidence interval [CI; 1.003–1.011]; *P* = 0.002; Table 2). After multivariable adjustment for clinical factors, laboratory findings, and comorbidities, PCT showed a significant association with AKI (OR 1.006, 95% CI [1.000–1.011]; *P* = 0.035; Table 2).

**Table 2:** Prognostic implication of PCT as a continuous variable on AKI development and 30-day mortality in critically ill patients.

	Crude	Model 1	Model 2	Model 3	Model 4
	OR (95% CI), *P*	OR (95% CI), *P*	OR (95% CI), *P*	OR (95% CI), *P*	OR (95% CI), *P*
AKI (N = 266)	1.007 (1.003–1.011) 0.002	1.007 (1.002–1.011), 0.003	1.008 (1.003–1.012), 0.001	1.006 (1.002–1.011), 0.007	1.006 (1.000–1.011), 0.035
30-day mortality (N = 99)	0.997 (0.990–1.004) 0.373	0.996 (0.989–1.003) 0.301	0.997 (0.989–1.004), 0.350	0.996 (0.989–1.003), 0.265	0.999 (0.991–1.007), 0.798

Model 1: adjusted for demographics (age >65, female sex).

Model 2: adjusted for demographics, and comorbidities (model 1 + CKD, DM, HTN, CVD, CLD, COPD, and asthma).

Model 3: adjusted for demographics, comorbidities, and AKI risk factors (model 2 + RAS blockers, inotropes, transfusion, ventilator, aminoglycosides, vancomycin, colistin, amphotericin, NSAID, contrast media, and chemotherapy).

Model 4: adjusted for demographics, comorbidities, AKI risk factors, and laboratory findings (model 3 + Hb, hsCRP, albumin, WBC, and SOFA score).

Abbreviations: OR, odds ratio; PCT, procalcitonin; AKI, acute kidney injury; CKD, chronic kidney disease; DM, diabetes mellitus; HTN, hypertension; CVD, cardiovascular disease; CLD, chronic liver disease; COPD, chronic obstructive pulmonary disease; RAS, renin-angiotensin-aldosterone system; CCB, calcium channel blocker; NSAID, nonsteroidal anti-inflammatory drug; hsCRP, highly sensitive C-reactive protein; WBC, white blood cell; SOFA, Sequential Organ Failure Assessment.

### Risk factors for 30-day mortality

Of the 790 patients used for total analysis, 99 patients died within 30 days of observation. Of the patients with 30-day mortality, 32 patients were in the sepsis group (67 patients were in the nonsepsis group). The mortality results from the multivariable Cox proportional regression analyses (using the same variables as model 4 of AKI) with serum PCT level as a continuous variable demonstrated that the hazard ratio (HR) of PCT for all-cause mortality was 0.999 (95% CI, 0.991–1.007, *P* = 0.798). The HR of mortality with a cut-off value of PCT >0.315 ng/ml used in the AKI prediction was 0.968 (95% CI, 0.586–1.597; *P* = 0.898). These results were not significant in either the sepsis group (HR, 1.991; 95% CI, 0.695–5.702: *P* = 0.200) or the nonsepsis group (HR, 0.876; 95% CI, 0.474–1.618: *P* = 0.200).

### Correlation analyses between PCT and other variables

Correlation analyses showed a positive correlation between PCT and hsCRP (ρ = 0.428, *P* < 0.001), AKI (ρ = 0.116, *P* = 0.001), and sepsis (ρ = 0.130, *P* < 0.001, Table 3).

**Table 3:** Cross-sectional correlation analyses between PCT and other variables.

	PCT	hsCRP	Age	Sex	AKI	Sepsis	Mortality
**PCT**							
ρ	1	0.428	0.107	0.028	0.116	0.130	0.054
*P*	—	<0.001	0.003	0.437	0.001	<0.001	0.131
**hsCRP**							
ρ		1	0.241	0.033	0.072	0.278	0.065
*P*		—	<0.001	0.359	0.042	<0.001	0.069
**Age**							
ρ			1	0.131	0.087	0.164	0.001
*P*			—	<0.001	0.014	<0.001	0.967
**Sex**							
ρ				1	0.011	0.039	−0.059
*P*				—	0.765	0.273	0.097
**AKI**							
ρ					1	0.088	0.012
*P*					—	0.014	0.730
**Sepsis**							
ρ						1	0.024
*P*						—	0.505
**Mortality**							
ρ							1
*P*							—

Abbreviations: PCT, procalcitonin; hsCRP, highly sensitive C-reactive protein; AKI, acute kidney injury.

### Determining diagnostic value of PCT in predicting AKI

We attempted to adjust the predictive cut-off value of PCT at varying levels. In Table 4, sensitivity, specificity, AUC, and likelihood ratio of PCT values for the prediction AKI at cut-off values of clinical condition (0.05, 0.5, 2, 10 ng/ml) were demonstrated. All thresholds were inadequate for clinical applications due to low sensitivity, specificity and AUC. Therefore, we tried to find the optimal threshold by calculation using the Youden method. In this study population, the cut-off value for PCT (0.315 ng/ml) obtained by the receiver operating characteristic (ROC) curve analysis was calculated as the optimal model for the prediction of AKI (AUC 0.589; sensitivity 60.9%; specificity 56.9%; Table 4).

**Table 4:** Summary of PCT values to predict AKI.

PCT (ng/ml)^a^	Clinical condition	PCT threshold (ng/ml)	AUC	Sensitivity	95% CI	Specificity	95% CI	+LR	−LR
<0.05	Healthy								
0.05~0.5	Local infection	0.05	0.561	0.744	0.688–0.796	0.378	0.336–0.421	1.196	0.677
0.5~2.0	Systemic infection (sepsis)	0.5	0.573	0.549	0.487–0.610	0.597	0.554–0.640	1.362	0.755
2.0~10	Severe sepsis	2.0	0.549	0.380	0.318–0.437	0.721	0.681–0.759	1.362	0.860
>10	Septic shock	10	0.544	0.229	0.180–0.285	0.863	0.830–0.891	1.672	0.893
Cut-off value^b^		0.315	0.589	0.609	0.548–0.668	0.569	0.525–0.612	1.413	0.687

Abbreviations: PCT, procalcitonin; AUC, area under curve; +LR, positive likelihood ratio; −LR, negative likelihood ratio. ^a^Commonly used PCT threshold in clinical practice. ^b^Threshold developed using the Youden method.

In the univariate analysis, PCT >0.315 ng/ml at the time of admission was significantly associated with the development of AKI (OR: 2.054, 95% CI: 1.520–2.775]; *P* < 0.001; Table 5). After multivariable adjustment for clinical factors, laboratory findings, and comorbidities, PCT still showed a significant association with AKI (OR 1.912, 95% CI [1.299–2.814]; *P* = 0.001; Table 5). Furthermore, we analyzed the relationship between PCT and AKI in each group with and without sepsis. In the sepsis group, PCT >0.315 ng/ml at the time of admission remained a significant independent risk factor for AKI (OR 2.422, 95% CI [1.222–4.802]; *P* = 0.011). In the nonsepsis group, the serum PCT level was also related to the development of AKI (OR 1.798, 95% CI [1.101–2.937]; *P* = 0.019; Table 5).

**Table 5:** Prognostic implication of PCT as a cut-off value on AKI development in patients with or without sepsis.

		Crude	Model 1	Model 2	Model 3	Model 4
		OR (95% CI), *P*	OR (95% CI), *P*	OR (95% CI), *P*	OR (95% CI), *P*	OR (95% CI), *P*
Total (N = 790)	PCT >0.315 ng/ml (N = 388)	2.054 (1.520–2.775) <0.001	2.035 (1.498–2.766), <0.001	2.069 (1.481–2.890), <0.001	2.019 (1.419–2.875), <0.001	1.912 (1.299–2.814), 0.001
No sepsis (N = 507)	PCT >0.315 ng/ml (N = 207)	1.751 (1.195–2.567) 0.004	1.761 (1.195–2.597) 0.004	1.754 (1.140–2.697), 0.011	1.692 (1.071–2.674), 0.024	1.798 (1.101–2.937), 0.019
Sepsis (N = 283)	PCT >0.315 ng/ml (N = 181)	2.405 (1.419–4.079) 0.001	2.372 (1.389–4.049) 0.002	2.634 (1.473–4.711), 0.001	3.031 (1.577–5.26), 0.001	2.422 (1.222–4.802), 0.011

Model 1: adjusted for demographics (age >65, female sex).

Model 2: adjusted for demographics, and comorbidities (model 1 + CKD, DM, HTN, CVD, CLD, COPD, and asthma).

Model 3: adjusted for demographics, comorbidities, and AKI risk factors (model 2 + RAS blockers, inotropes, transfusion, ventilator, aminoglycosides, vancomycin, colistin, amphotericin, NSAID, contrast media, and chemotherapy).

Model 4: adjusted for demographics, comorbidities, AKI risk factors, and laboratory findings (model 3 + Hb, hsCRP, albumin, WBC, and SOFA score).

Abbreviations: OR, odds ratio; PCT, procalcitonin; AKI, acute kidney injury; CKD, chronic kidney disease; DM, diabetes mellitus; HTN, hypertension; CVD, cardiovascular disease; CLD, chronic liver disease; COPD, chronic obstructive pulmonary disease; RAS, renin-angiotensin-aldosterone system; CCB, calcium channel blocker; NSAID, nonsteroidal anti-inflammatory drug; hsCRP, highly sensitive C-reactive protein; WBC, white blood cell; SOFA, Sequential Organ Failure Assessment.

**Figure 2:**
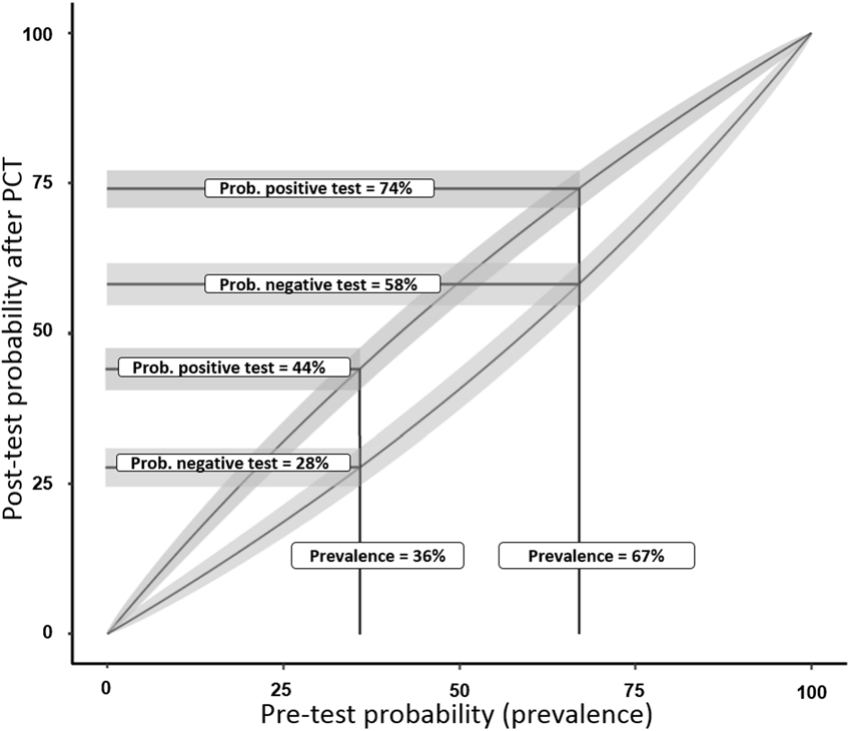
Pre- and posttest probability after PCT for AKI development in critically ill patients. Posttest probability was calculated based on pretest probability and the likelihood ratio: posttest odds = pretest odds × likelihood ratio. The graph shows that for a patient with a pretest probability of AKI of 36~67%, the posttest probability will be 44~74% following a positive test result (PCT >0.315 ng/ml) and 28~58% following a negative test result (PCT ≤ 0.315 ng/ml).

To assess the diagnostic usefulness of PCT, we examined the differences between pre- and posttest probability. The likelihood ratio of a positive test result [sensitivity/(1-specificity)] was 1.413, and the likelihood ratio of a negative test result [(1-sensitivity)/specificity] was 0.687. Using the 36~67% prevalence of AKI in critically ill patients from previous reports^2–4^, we estimated the probabilities of AKI to be 44~74% following a positive test result (PCT >0.315 ng/ml) and 28~58% following a negative test result (PCT ≤ 0.315 ng/ml). Interactive graphical presentation was illustrated by the Clinical Accuracy and Utility tool by Allen *et al*.^14^ (Fig. 2).

## Discussion

This study does not support the usefulness of adding PCT levels to existing models for predicting the occurrence of AKI in critically ill patients in the ICU. The PCT value was higher in the AKI group, and it should be noted that careful attention is needed to interpret the clinical significance of PCT in critically ill patients with reduced renal function. However, despite the association between PCT and AKI, we were unable to establish the complete the enhancement of AKI prediction by adding PCT to the existing AKI risk factors.

The kinetics of PCT, including the elimination route or mechanism, are not fully known. However, considering its low molecular weight of 13,600 Da, impaired renal function is thought to affect PCT levels. In previous studies, end-stage renal disease patients had a mean serum PCT value of 0.69 ± 0.81 ng/ml before dialysis and presented higher than the standard value of 0.5 ng/ml in 57% of patients^15^. The mean serum PCT level decreased significantly to 83 ± 25% after high-flux dialysis, whereas no change in PCT concentration occurred after low-flux dialysis^11^. In addition, PCT was elevated in patients on continuous ambulatory peritoneal dialysis (median of 1.18 ng/ml)^12^. Several studies have demonstrated a significant positive correlation between PCT and creatinine and a significant negative correlation between PCT and estimated glomerular filtration rate (eGFR)^10,12,16^. These findings suggest that PCT is partially removed via the kidney and is therefore dependent on renal function. The results of the current investigation also imply that PCT may be influenced by renal function. According to existing research^13,16,17^, the diagnostic accuracy of PCT showed significantly lower sensitivity and specificity to the reliability of bacterial infections in patients with renal impairment. Because renal function is a major determinant of PCT levels, higher cut-off values should be applied to patients with impaired renal function^18^.

PCT is recognized as a suitable marker for the diagnosis of sepsis or severe sepsis. However, other noninfective conditions, such as surgery, trauma, burn, pancreatitis, and renal dysfunction, can also increase serum PCT levels. These causes lead to ICU admittance and extensive tissue trauma, ischemia, and reperfusion. Moreover, similar to sepsis, high levels of catecholamines (stress hormones) can activate inflammation cascades^19^. In some of our patients, PCT was elevated above the cut-off value (PCT >0.315 ng/ml) despite their nonsepsis status, and the following cases were common: postcardiac arrest syndrome, pancreatitis & cholangitis, acute decompensated heart failure and drug intoxication. In addition, there is a possibility that the PCT level may be affected by differences in comorbidities such as CKD, DM, and HTN between AKI and non-AKI patients. In addition, the discrepancy in the frequency of comorbidities between the AKI and non-AKI groups may also contribute to differences in PCT levels, regardless of sepsis.

The pathophysiological mechanism explaining the relationship between serum concentrations of PCT and AKI is still unclear. Various inflammatory responses have been suggested as a possible mechanism for the development of AKI^20,21^. Experimental data have confirmed that bacterial toxins and other mediators can induce PCT^22^. PCT acts as a chemoattractant in the inflammatory area, directly attracting more monocytes. PCT is initially produced in adherent monocytes that later contribute to the marked increase in circulating PCT by recruiting parenchymal cells when they are in direct contact with activated monocytes. The expression of PCT mRNA by peripheral blood mononuclear cells is stimulated by LPS or other toxins released by microbes^23–26^. Additionally, it is suggested that an indirect pathway induced through cell-mediated host responses caused by inflammatory cytokines (e.g., IL-1β, IL-6, and TNF-α) plays a pivotal role in AKI^24,27^. Another possible mechanism represents the direct toxic effect of PCT on mesangial cells^22^. This study showed that mesangial cells could be destroyed by PCT through increased synthesis of IL-6, iNOS, and TNF-α, inducing disruption of actin microfilaments and apoptosis. This result reflects the possibility of PCT as a toxic mediator of AKI. In some studies^12,13^, PCT elevations have been shown to affect impaired renal function, but there is a lack of evidence as to whether PCT can be used as a predictive marker for AKI. Based on the above-described mechanism, we analyzed the diagnostic value of AKI by dividing the patient group according to various levels of PCT (PCT: 0.05, 0.5, 2, 10 ng/ml), which was not clinically useful. In addition, when calculating the predictive cut-off value (PCT >0.315 ng/ml) and analyzing the results, the association between the PCT cut-off value and subsequent AKI development was significant regardless of sepsis. However, the posttest probability did not show a significant improvement. Therefore, if a critically ill patient exhibits PCT elevation when an AKI occurs during treatment, other causes should be considered rather than a change due to kidney dysfunction.

Unlike other studies^28^, elevations in PCT were not associated with mortality in our study. In a study comparing mortality by serial PCT measurement^29^, mortality at day 28 increased by two fold in contrast to mortality at day 4, when PCT did not decrease by more than 80%. Our study did not conclude that PCT and mortality were statistically correlated, even though the number of patients who died before 30 days was high. This may be likely due to the PCT levels and the high severity and comorbidity of the patients upon admission. This hypothesis is difficult to confirm because our study measured PCT once at admission. There was no difference in the SOFA score between the AKI and non-AKI groups (Table 1), and the score was similar when we divided the groups according to the presence or absence of sepsis and death (data not shown). We assume that the reason for the lack of correlation between PCT and mortality is that the severity of illness does not vary with mortality, which needs to be verified through serial measurement of PCT.

The study has several limitations. First, this is a retrospective study involving a small sample size in a single center. Second, we did not examine other AKI biomarkers, such as NGAL or cystatin C. Therefore, it is not possible to determine the diagnostic accuracy of PCT in comparison to another AKI biomarker. Third, we only examined PCT levels upon ICU admission and therefore cannot address the variability in inflammatory markers during the course of the study. To fully understand the relationship between changes in PCT levels and patient prognosis, sequential blood sampling may be needed to investigate the effectiveness of therapeutic strategies and the prognostic role of PCT.

In summary, serum PCT levels were significantly higher in patients with AKI than in those without AKI, and the most appropriate PCT cut-off value was calculated and analyzed. There was an association between PCT and AKI, but it was insufficient to provide diagnostic usefulness. Therefore, the use of PCT for the clinical diagnosis of sepsis in patients with decreased renal function requires more caution than usual, and the elevation of PCT in patients with AKI may be necessary to identify other infective causes other than renal dysfunction.

In conclusion, this study suggests that the PCT value was higher in AKI patients than in non-AKI patients, but the PCT measurement at the time of admission did not improve the prediction model for AKI in critically ill patients.

## Methods

### Study design and participants

This study was a retrospective study to determine the role of PCT in the development of AKI in critically ill patients in the ICU. From January 2009 to December 2013, ICU inpatients in Gachon University Gil Medical Center who had PCT levels measured at the time of admission were analyzed. A total of 12,051 patients were examined. Patients whose age was 18 years or younger (N = 2,321), who were transferred to the ICU after initial hospitalization (N = 7,254), who had already received dialysis before the PCT measurement (N = 991), or whose timing of the PCT measurements was not consistent with baseline parameter measurements (N = 695) were excluded from the study. Therefore, 790 patients were finally analyzed. This study was approved by the Institutional Review Board (IRB) of our institution (GAIRB2016-086). Because this study did not involve any further intervention in the retrospective analysis, the need for obtaining consent was waived by the IRB.

### Variables

Demographics (age, sex, comorbidities, and prescribed medication) and blood test results (blood gas analysis, white blood cell count [WBC], levels of hemoglobin [Hb], hematocrit [Hct], platelets, blood urea nitrogen [BUN], creatinine, albumin, and highly sensitive C-reactive protein [hsCRP]) were recorded as baseline data at the time of admission. Clinical data (mechanical ventilation, sepsis status at admission, use of inotropes, and blood transfusion) and comorbidities (history of CKD, DM, pre-existing HTN, CVD, CLD, asthma, and chronic obstructive pulmonary disease [COPD]) prior to ICU admission treatment were recorded according to the ICD-10 diagnostic criteria. The use of drugs with potential renal toxicity (e.g., angiotensin converting enzyme inhibitors, angiotensin II receptor blockers, nonsteroidal anti-inflammatory drugs), the use of contrast media within 24 hours of developing AKI, and the use of anticancer drugs with a platinum-based regimen within the last 6 months were also investigated. Severity of illness was assessed using the Sequential Organ Failure Assessment (SOFA) scoring system^30^.

The eGFR was calculated according to the four-variable Modification of Diet in Renal Disease (MDRD) Study formula^31^. Cardiovascular risk factors were assessed at the beginning of the observation. Patients were classified according to the ICD-10 code for baseline disease. In addition, hypertension was defined as a blood pressure of >140/90 mmHg measured at least 2 times and a prescription of antihypertensive agents. CVD history was defined as any previous myocardial infarction, angina pectoris, ischemic heart disease, cerebrovascular accident, atrial fibrillation or flutter, or peripheral atherosclerotic vascular disease. DM was defined as the use of an oral anti-diabetic agent or insulin. CKD was defined as having evidence of kidney damage (proteinuria estimated by dipstick as trace or greater) with underlying disease or eGFR less than 60 mL/min/1.73 m^2^ ^32^.

### Outcomes

The primary outcome was the development of AKI. We followed the Kidney Disease: Improving Global Outcomes (KDIGO) clinical practice guidelines for the diagnosis and classification of AKI, which classifies renal damage due to AKI based on serum creatinine levels^33^. The baseline serum creatinine was measured at the time of admission. The diagnostic criteria for AKI were defined as an absolute increase in serum creatinine value ≥ 0.3 mg/dl or a 50% relative increase from baseline value within 48 hours. The risk factors for AKI were also assessed at the time of ICU admission. The secondary outcome was 30-day mortality rate. The diagnosis of sepsis (including severe sepsis and septic shock) was defined by the American college of chest physicians/society of critical care medicine (ACCP/SCCM) consensus conference committee^34^.

### Statistical analysis

The data are presented as the mean ± standard deviation for continuous variables and as proportions for categorical variables. Variables with nonnormal distributions are expressed as the medians [interquartile range (IQR)] based on variable distributions using histograms and Kolmogorov-Smirnov test. A chi-squared test was used to compare categorical variables. Comparisons between the normally and nonnormally distributed continuous variables were performed using Student’s t-tests and Mann-Whitney *U* tests, respectively.

We first analyzed the diagnostic value of PCT as a continuous variable and then determined the PCT cut-off level for predicting AKI. We used the Youden index of the ROC curve^35^. The risk factors associated with AKI were analyzed using multivariable logistic regression analyses to obtain the odds ratio (OR) and 95% confidence interval (CI). Multiple Cox regression models were used to analyze the risk factors associated with 30-day mortality and calculate the HRs. As a stepwise regression model, we applied the method used in our previous study^36^ and analyzed it as follows. First, the model was performed using demographic variables only (model 1). Second, comorbidities were added to model 1 for model 2 analysis. Third, we analyzed the AKI risk factors in addition to model 2 to create model 3, and finally, we added laboratory data and SOFA scores to model 3 to create model 4.

In addition, we attempted to determine whether there is a clinical value worth considering in the diagnosis according to the results of a previous study^37^. The diagnostic values were evaluated by comparing the pre- and postprobabilities of the analysis results with the model containing only existing risk factors and the model with PCT added.

Differences at *P* < 0.05 were considered statistically significant. For the analysis, commercially available SPSS 20.0 software (IBM SPSS Inc., Armonk, NY, USA) and MedCalc 17.2 (MedCalc Software, Ostend, Belgium) were used.
